# Differences in serum and plasma levels of microRNAs and their time-course changes after blood collection

**DOI:** 10.1016/j.plabm.2024.e00376

**Published:** 2024-02-16

**Authors:** Ichiro Wakabayashi, Mikio Marumo, Kazumi Ekawa, Takashi Daimon

**Affiliations:** a Department of Environmental and Preventive Medicine, School of Medicine, Hyogo Medical University, Nishinomiya, Hyogo, 663-8501, Japan; b Department of Biostatistics, School of Medicine, Hyogo Medical University, Nishinomiya, Hyogo, 663-8501, Japan

**Keywords:** Biomarkers, Erythrocytes, microRNA, Plasma, Platelets, Serum

## Abstract

**Background:**

Serum and plasma are used for measurements of microRNAs (miRNAs) as biomarkers of various diseases. However, no consistent findings have been obtained regarding differences in serum and plasma levels of miRNAs. The purpose of this study was to clarify differences in serum and plasma levels of total miRNAs and their time-course changes after blood collection.

**Methods:**

Venous blood was collected from healthy men, and samples were prepared at the time points of 0, 15, 30, 60 and 180 min after blood collection for plasma and after clot formation for serum. Levels of total miRNAs were analyzed by the hybridization method using the 3D-Gene miRNA Oligo chip.

**Results:**

About one third of 2632 miRNAs tested showed levels high enough for comparison of serum and plasma levels and for investigation of their time-course changes. Levels of 299 miRNAs at time 0 were significantly different in serum and plasma. Levels of representative platelet-derived miRNAs including miR-185-5p, -22-3p and -320b were significantly higher in plasma than in serum, while levels of representative erythrocyte-derived miRNAs including miR-451a, -486-5p and -92a-3p were not significantly different in serum and plasma. Plasma levels of 173 miRNAs and 6 miRNAs showed significant decreasing and increasing tendencies, respectively, while there were no miRNAs in serum that showed significant time-course changes.

**Conclusion:**

The results suggest that careful attention should be paid when comparing serum and plasma levels of miRNAs and that plasma samples should be prepared early after blood collection.

## Highlights

• Levels of 299 miRNAs were significantly different in serum and plasma.
• Levels of several platelet-derived miRNAs were higher in plasma than in serum.
• Several erythrocyte-derived miRNA levels were not different in serum and plasma.
• Plasma levels of 173 miRNAs tended to decrease with time after blood collection.
• There were no miRNAs in serum that showed significant time-course changes.

## 1 Introduction

MicroRNAs (miRNAs) are small non-coding RNAs and act as post-transcriptional gene silencers through interacting with specific messenger RNAs. miRNAs are present in various biofluids, including blood, urine, saliva, tears, seminal fluid, amniotic fluid, bronchial lavage, cerebrospinal fluid, pleural fluid, peritoneal fluid, colostrum and breast milk [1], and have been proposed to be useful as diagnostic and prognostic biomarkers and predictors of drug response [2]. Serum and plasma are used as blood-derived biofluids for measurement of miRNAs. However, there have been no consistent findings regarding the difference in miRNA levels in serum and plasma [[3], [4], [5], [6]]. Moreover, to the best of our knowledge, there has been no report on differences in miRNA levels in serum and plasma by analyzing total miRNAs.

Blood clot formation is necessary for serum separation and thus blood coagulation is included in the process of serum preparation, while blood coagulation is blocked by an anticoagulant in plasma preparation. Thus, levels of blood coagulation-related miRNAs, which are mainly derived from platelets, are possibly affected during serum preparation. In addition, erythrocytes are also contained in blood clots and are possibly affected by clot formation. Erythrocyte-derived miRNAs are released by hemolysis, which has been shown to affect the miRNA profile of blood [7]. Moreover, strong associations have been shown among serum levels of erythrocyte-derived miRNAs [8].

The purpose of this study was therefore to clarify the differences in serum and plasma levels of total miRNAs, including erythrocyte- and platelet-derived miRNAs. We also investigated whether levels of each miRNA in serum and plasma showed time-dependent changes after clot formation and blood collection, respectively. The above information would be useful for clinical and epidemiological studies on miRNAs.

## 2 Methods

### 2.1 Participants

Participants were healthy male Japanese (n = 5) who were not receiving any medications. Their mean age with standard deviation was 37.2 ± 10.4 years. All of the participants were nonsmokers. The protocol of this study was approved by the Hyogo College of Medicine Ethics Committee (No. 3036 in 2020). Written informed consent was provided by all of the participants.

### 2.2 Blood sample collection

Blood was collected from each participant after overnight fasting, and serum and plasma were prepared as follows. For serum preparation, the whole blood was collected in an evacuated plastic tube containing silica-coated film (VP-AS074K, Terumo Corp., Tokyo, Japan), and the blood was allowed to clot by leaving it undisturbed at room temperature for 30 min. Then, the clot was removed by centrifugation at 2300×*g* for 10 min. For plasma preparation, the whole blood was collected in a plastic tube containing EDTA-2Na (3 mg per 2 ml of whole blood, VP-NA052K, Terumo Corp., Tokyo, Japan) as an anticoagulant, and cells were removed by centrifugation at 1200×*g* for 10 min. For time-course experiments, each plasma sample was prepared at 0, 15, 30, 60 and 180 min after blood collection, and each serum sample was prepared at 0, 15, 30, 60 and 180 min after clot formation for 30 min. The samples were kept frozen at -80° until analyses of miRNAs.

### 2.3 RNA extraction and miRNA expression profiling

RNA was extracted from a serum or plasma sample (300 μl) using 3D-Gene RNA extraction reagent from a liquid sample kit (Toray Industries Inc., Kamakura, Japan) according to the manufacturer's instructions and extracted total RNA was checked by Bioanalyzer (Agilent, CA, USA). miRNA expression was analyzed by the hybridization method using the 3D-Gene miRNA Oligo chip (TRT-XR520, Toray) and 3D-Gene miRNA labeling kit (TRT-XE211, Toray) as described previously [9]. Briefly, half volumes of labeled RNAs were hybridized onto a 3D-Gene miRNA Oligo chip (Toray), which was designed to detect sequences of multiple miRNAs, and the annotation and oligonucleotide sequences of the probes were conformed to the miRBase release 21, miRNA database (http://microrna.sanger.ac.uk/sequences/). After stringent washes, fluorescent signals were scanned with a 3D-Gene Scanner (Toray) and analyzed using 3D-Gene Extraction software (Toray). miRNA expression was normalized as follows. The raw data of each spot were substituted with a mean intensity of the background signal determined by signal intensities of all blank spots with signal intensity of the top and bottom 5% (out of 95% confidence intervals) being removed. Measurements of spots were considered to be valid when the signal intensities were greater than 2 standard deviations of the background signal intensity. The signal intensities of the valid spots were compared and a relative expression level of a given miRNA was calculated throughout the microarray experiments. Global normalization of the data was performed for each array, so that the median of the signal intensity was adjusted to 25.

### 2.4 Statistical analysis

Each miRNA level was transformed with the logarithm to the base 2. Fold change in the mean miRNA level of plasma versus serum was calculated as the ratio of the inverse logarithm with the base 2 of the mean of the plasma miRNA levels to that of the mean of the serum miRNA levels. The plasma and serum miRNA levels are summarized as means with standard errors (Table 1) and were compared with the use of Student's t-test. Time-dependent changes in the mean miRNA levels for plasma and serum were assessed with the use of repeated-measures analysis of variance (ANOVA). For each of the miRNAs, on the basis of the observed distribution of p values, we estimated q values (the proportion of false positives incurred, called the false discovery rate), according to the method of Storey et al. [10]. All p and q values were two-sided. A q-value of less than 0.05 was considered statistically significant. Data were analyzed with the use of R version 4.0.3 [11] and SPSS version 22.0 Armonk, NY, USA.

**Table 1:** Comparisons of erythrocyte- and platelet-derived miRNA levels in serum and plasma and their time-course changes.

	miRNA levels^a^				Time-course change	
	plasma	serum	q value	fold (plasma/serum)	plasma	serum
**Erythrocytes**						
**miR-25-3p**	4.54 ± 0.27	3.68 ± 0.10	0.083	1.82	n.d.	n.d.
**miR-423-5p**	7.26 ± 0.15	7.74 ± 0.09	0.027	0.72	q = 0.001	q = 0.763
**miR-451a**	9.39 ± 0.52	8.48 ± 0.31	0.260	1.88	q = 0.053	q = 0.763
**miR-486-5p**	6.71 ± 0.30	6.17 ± 0.27	0.202	1.45	q = 0.051	q = 0.763
**miR-92a-3p**	6.38 ± 0.19	5.59 ± 0.19	0.089	1.73	q = 0.037	q = 0.763
**Platelets**						
**let-7b-5p**	4.12 ± 0.38	2.64 ± 0.28	0.073	2.79	n.d.	n.d.
**miR-17-5p**	5.34 ± 0.32	3.88 ± 0.18	0.060	2.74	n.d.	n.d.
**miR-185-5p**	4.73 ± 0.14	3.54 ± 0.18	0.024	2.27	q = 0.087	q = 0.793
**miR-22-3p**	6.72 ± 0.12	5.60 ± 0.21	0.009	2.17	q = 0.045	q = 0.763
**miR-24-3p**	8.56 ± 0.21	7.06 ± 0.70	0.078	2.84	q = 0.064	q = 0.726
**miR-320b**	5.63 ± 0.05	4.95 ± 0.13	0.026	1.60	q = 0.052	q = 0.620
**Erythrocytes & platelets**						
**miR-16-5p**	6.55 ± 0.48	4.59 ± 0.49	0.077	3.89	n.d.	n.d.

a Shown are means with standard errors of base-2 log-transformed values of intensities of miRNAs in plasma and serum. n.d.: not determined.

## 3 Results & discussion

### 3.1 Comparisons of levels of total miRNAs in serum and plasma

**Fig. 1:**
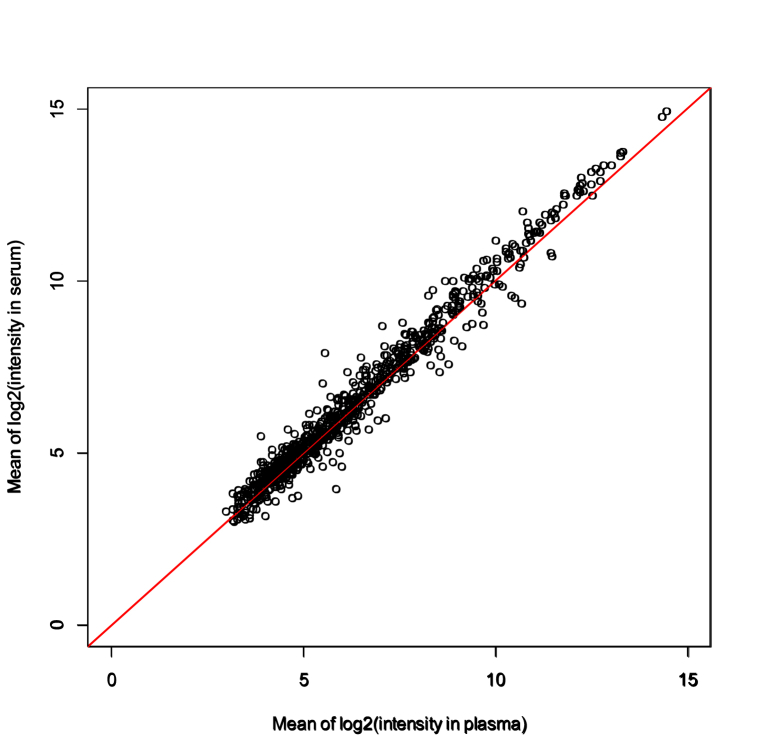
Scatter plots of mean plasma and serum levels of 985 miRNAs after base-2 logarithmic transformation.

Among 2632 miRNAs tested, levels of 985 miRNAs were high enough for comparison of their levels in serum and plasma samples (just after blood collection for plasma and just after clot formation for 30 min for serum), and levels of 823 miRNAs were high enough for comparison at five different time points after blood collection for plasma or after clot formation for serum. Scattered plots of 985 miRNAs in serum and plasma are shown in Fig. 1. All of the information on differences in serum and plasma levels of total miRNAs are given in Supplementary Table 1. There were significant differences in serum and plasma levels of about one-third of the miRNAs (n = 299) at time 0. Serum levels of 249 miRNAs were significantly higher than their plasma levels, while plasma levels of 50 miRNAs were significantly higher than their serum levels. Some of these differences were speculated to be due to clot formation during preparation of serum since miRNA-containing blood cells are included in the clot.

### 3.2 Comparisons of levels of erythrocyte- and platelet-derived miRNAs in serum and plasma

Levels of erythrocyte- and platelet-derived miRNAs in serum and plasma at time 0 were then compared (Table 1). We selected five erythrocyte-derived miRNAs (miR-25-3p, -423-5P, -451a, -486-5p and -92a-3p), six platelet-derived miRNAs (let-7b-5p, miR-17-5p, -185-5p, -22-3p, -24-3p and -320b) and one erythrocyte- and platelet-derived miRNA (miR-16-5p) for comparison [[12], [13], [14]]. Regarding platelet-derived miRNAs, levels of miR-185-5p, -22-3p and -320b were significantly higher in plasma than in serum. Levels of the other platelet-derived miRNAs (let-7b-5p, miR-17-5p and -24-3p) were not significantly different in serum and plasma but tended to be higher in plasma than in serum (fold change [plasma/serum]: >2). Similarly, levels of miR-16-5p, which is derived both from erythrocytes and platelets, were not significantly different (q = 0.077) in serum and plasma but tended to be higher in plasma than in serum (fold change: 3.89). The above results of comparisons of platelet-derived miRNAs were rather unexpected since they were supposed to be released by activation of platelets during blood coagulation when serum was prepared. In fact, it has been shown that antiplatelet therapy decreased platelet-derived miRNA levels in plasma [15]. A possible reason for the higher levels of the platelet-derived miRNAs in plasma than in serum is that exosomes containing platelet-derived miRNAs in blood were absorbed in the clot during preparation of serum and/or released from platelets during plasma sample preparation. However, there is a discrepancy in the results of previous studies regarding other platelet-derived miRNAs including let-7b-5p, miR-126, -199a-5p and -223 in serum and plasma: Levels of miR-126 and -223 were reported to be higher in plasma than in serum [4], while the level of let-7b-5p was higher in serum than in plasma [6]. Moreover, levels of miR-223 and -199a-5p were reported to be higher in serum than in plasma [5]. Possible reasons for the discrepancy in the results of those studies are differences in experimental conditions including the method used for measurement of miRNA and the timing of sample preparation after blood collection as described below.

On the other hand, there were no significant differences in serum and plasma levels of representative erythrocyte-derived miRNAs including miR-25-3p, -451a, -486-5p and -92a-3p. These results imply that hemolysis did not occur during serum preparation and exosomes containing erythrocyte-derived miRNAs in blood were not captured in the clot during serum preparation. The level of miR-423-5p, another erythrocyte-derived miRNA, was significantly higher in serum than in plasma. There is also a discrepancy regarding levels of the representative erythrocyte-derived miRNAs, miR-451 and -486, in serum and plasma. The level of miR-451 was higher in serum than in plasma in two studies [3,4], while it was lower in serum than in plasma in another study [5]. The level of miR-486 was higher in plasma than in serum in one study [5], whereas its levels were comparable in serum and plasma in another study [6]. In the present study, there was a considerable number of other miRNAs that showed significantly higher levels in serum than in plasma, for which the reason remains unknown.

### 3.3 Time-course changes in levels of total miRNAs in serum and plasma

**Fig. 2:**
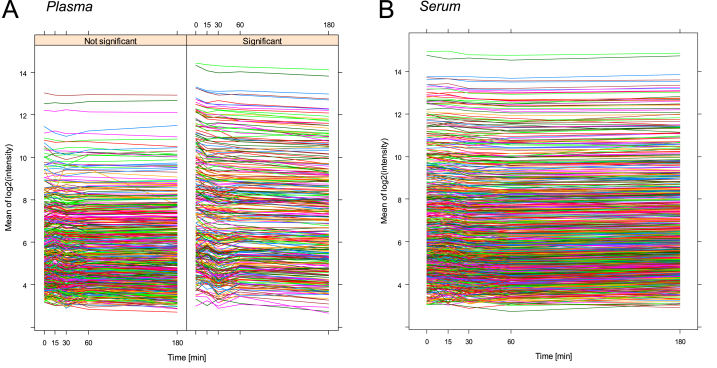
Time courses of mean plasma and serum levels of 823 miRNAs after base-2 logarithmic transformation. Levels of each of the 823 miRNAs were compared at five time points (0, 15, 30, 60 and 180 min) after blood collection for plasma (**A**) and after clot formation for serum (**B**). The plasma levels of miRNAs with and without significant time-dependent changes are separately displayed (**A**).

All of the information on time-course changes in serum and plasma levels of total miRNAs are given in Supplementary Table 2. Significant changes in the levels of 305 miRNAs (37%) in plasma were observed during the time course (0–3 h) after blood collection. Plasma levels of 173 miRNAs tended to decrease with time, while plasma levels of 6 miRNAs (miR-128-2-5p, -3619-3p, -3620-3p, -4649-5p, -4758-3p and -6885-5p) tended to increase with time (Fig. 2A, Supplementary Table 2). The reason for the time-dependent decreases in 21% of the total miRNAs in plasma may be endocytosis of miRNA-containing exosomes by blood cells. Therefore, the time after blood collection till plasma sample preparation should be similar when miRNAs in the samples are compared. Early preparation of plasma samples of miRNAs is thought to be better for evaluation of their in-vivo status in blood.

On the other hand, no significant time-dependent changes were observed in serum levels of all (n = 823) miRNAs (Fig. 2B, Supplementary Table 2), and this finding suggests that more stable results of miRNA levels are obtained by using serum than by using plasma as a blood-derived biofluid. As a reason for no significant time-dependent changes in serum levels of all of the miRNAs tested, there was a possibility of higher variability of serum levels of each miRNA than that of plasma levels since the process of clot formation is included in the process of serum preparation. However, this hypothesis is unlikely because of similar relationships between mean values and standard errors of miRNA levels in serum and plasma presented in Table 1.

### 3.4 Time-course changes in levels of erythrocyte- and platelet-derived miRNAs in serum and plasma

**Fig. 3:**
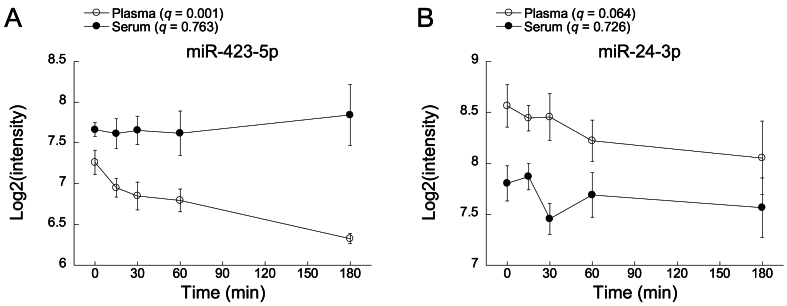
Time courses of levels of miR-423-5p (A) and miR-24-3p (B) in plasma and serum.

Table 1 also shows time-course changes in erythrocyte- and/or platelet-derived miRNAs in serum and plasma. In plasma, levels of miR-423-5p, -92a-3p and -22-3p showed significant time-course changes, while levels of other miRNAs (miR-451a, -486-5p, -185-5p, -24-3p and -320b) did not show significant time-course changes. Although not significant, plasma levels of these five miRNAs tended to change with time (borderline significances with q values of 0.051–0.087). As shown in Fig. 3, plasma levels of miR-423-5p and -24-3p tended to decrease time-dependently. On the other hand, no significant time-dependent changes were found in the serum levels of all of the erythrocyte- and/or platelet-derived miRNAs tested (Table 1, Supplementary Table 2, Figs. 2B and 3). Regarding the time-course changes in miRNAs, analyses of miR-25-3p, let-7b-5p, miR-17-5p and 16-5p in serum and plasma were not performed (Table 1) because samples showing very low intensity levels were included in the data for analysis.

### 3.5 Clinical implications

Because of their high stability in blood, miRNAs are considered as promising biomarkers for diagnosis and prognosis of various diseases [16]. In the present study, there were no miRNAs in serum that showed significant changes during the time course (0–3 h) after blood collection, whereas 37% of the miRNAs in plasma showed significant time-course changes. Since it is clinically not easy to adjust the time of plasma or serum separation after blood collection, it is better to use serum clinically as a sample of circulating miRNAs. This recommendation is also supported by the following findings of this study: When serum was used as a sample of miRNAs, there were possibilities of influences on serum miRNA levels by hemolysis and blood coagulation. However, levels of erythrocyte-derived miRNAs were comparable in serum and plasma, and levels of platelet-derived miRNAs were not higher in serum than in plasma. These results suggest that neither hemolysis nor blood coagulation influenced miRNA levels in serum. On the other hand, there is a possibility of erroneous diagnosis when plasma samples prepared a while after blood collection are used for measurement of miRNAs.

### 3.6 Study limitation

There are limitations of this study. Serum and plasma levels of total miRNAs were measured by a direct hybridization method using a microarray chip. Thus, levels of about two thirds of the miRNAs tested were too low to perform statistical analysis. Therefore, measurement using PCR amplification is needed to compare those miRNAs in future studies. The sample size in this study was small and future studies using larger sample sizes are also needed to confirm the findings of the present study.

## 4 Conclusion

There were significant differences in serum and plasma levels of about one third of the miRNAs tested, and plasma levels of about one fifth of the miRNAs tended to decrease with time during the 3-h period after blood collection. These findings of differences in levels of miRNAs in plasma and serum samples and their time-dependent changes will be useful for clinical and epidemiological studies on miRNAs. The results of this study suggest that careful attention should be paid when comparing serum and plasma levels of miRNAs in different studies. Moreover, the timing of preparation of plasma samples should be early after blood collection.

## Funding

This study was supported by a Grant-in-Aid for Scientific Research (No. 21H03386) from the 10.13039/501100001691Japan Society for the Promotion of Science.

## CRediT authorship contribution statement

**Ichiro Wakabayashi:** Writing – original draft, Validation, Methodology, Investigation, Funding acquisition, Formal analysis, Data curation, Conceptualization. **Mikio Marumo:** Writing – review & editing, Methodology. **Kazumi Ekawa:** Writing – review & editing, Methodology. **Takashi Daimon:** Writing – review & editing, Methodology, Investigation, Data curation.

## Declaration of competing interest

None.

## Data Availability

Data will be made available on request.
